# Gene Duplication and Fragment Recombination Drive Functional Diversification of a Superfamily of Cytoplasmic Effectors in *Phytophthora sojae*

**DOI:** 10.1371/journal.pone.0070036

**Published:** 2013-07-29

**Authors:** Danyu Shen, Tingli Liu, Wenwu Ye, Li Liu, Peihan Liu, Yuren Wu, Yuanchao Wang, Daolong Dou

**Affiliations:** Department of Plant Pathology, Nanjing Agricultural University, Nanjing, China; Oregon State University, United States of America

## Abstract

*Phytophthora* and other oomycetes secrete a large number of putative host cytoplasmic effectors with conserved FLAK motifs following signal peptides, termed crinkling and necrosis inducing proteins (CRN), or Crinkler. Here, we first investigated the evolutionary patterns and mechanisms of CRN effectors in *Phytophthora sojae* and compared them to two other *Phytophthora* species. The genes encoding CRN effectors could be divided into 45 orthologous gene groups (OGG), and most OGGs unequally distributed in the three species, in which each underwent large number of gene gains or losses, indicating that the CRN genes expanded after species evolution in *Phytophthora* and evolved through pathoadaptation. The 134 expanded genes in *P. sojae* encoded family proteins including 82 functional genes and expressed at higher levels while the other 68 genes encoding orphan proteins were less expressed and contained 50 pseudogenes. Furthermore, we demonstrated that most expanded genes underwent gene duplication or/and fragment recombination. Three different mechanisms that drove gene duplication or recombination were identified. Finally, the expanded CRN effectors exhibited varying pathogenic functions, including induction of programmed cell death (PCD) and suppression of PCD through PAMP-triggered immunity or/and effector-triggered immunity. Overall, these results suggest that gene duplication and fragment recombination may be two mechanisms that drive the expansion and neofunctionalization of the CRN family in *P. sojae*, which aids in understanding the roles of CRN effectors within each oomycete pathogen.

## Introduction

Many pathogens secrete a large repertoire of effector molecules (virulence factors) that act within the cytoplasm of host cells to suppress host defenses and enhance microbial fitness [1]–[3]. These effectors may be recognized by plant disease resistance (R) proteins, resulting in effector-triggered immunity (ETI) to restrict or eliminate infecting pathogens [2]. The reciprocal selective pressures imposed by the interplay of pathogen effectors and host defense systems drive pathogens towards more virulent effectors or loss of the effectors recognized by R proteins, while concurrently selecting for plants with strengthened surveillance systems [4].

The genus *Phytophthora*, containing over 100 species, is a devastating oomycete phytopathogen that targets a wide variety of plants important to agriculture and forestry [5]. For example, *Phytophthora infestans*, the Irish potato famine pathogen, causes the devastating tomato and potato late blight disease [6]. *Phytophthora ramorum* is responsible for sudden oak death that affects live oaks and other woody shrubs [7]. Another species, *P. sojae*, causes soybean root and stem rot leading to losses of $1 to $2 billion per year worldwide [8]. *Phytophthora* is a fungus-like oomycete, which lacks taxonomic affinity with true fungi but is instead more closely related to diatoms and brown algae of the stramenopiles or heterokonts [8], [9]. Draft genome sequences have been completed for the above three *Phytophthora* species [6], [10].

*Phytophthora* species secrete a large number of RXLR effectors that are translocated inside host cells. RXLR effectors are modular proteins with two main functional domains. The N-terminal domain includes the signal peptide and conserved RXLR motif, and functions in the delivery of effectors into host cells [11], [12], while the C-terminal domain plays a role in virulence. RXLR effectors not only trigger ETI in plant genotypes possessing cognate R genes but also contribute to virulence by suppressing PTI and/or ETI [13]–[15]. Hundreds of potential RXLR effector genes are found in the genomes of sequenced *Phytophthora* species, and they show extensive diversity in their sequences with relatively rapid birth-and-death evolution [16]. Moreover, analysis of paralogous RXLR genes in *P. infestans* indicates that positive selection has played a major role in shaping the C-terminal region [17]. RXLR effector genes typically appear in gene sparse and repeat-rich regions of the genome, and mobile elements contributing to the dynamic nature of these repetitive regions may trigger recombination events, resulting in the increased rates of gene gain and gene loss observed for these effectors [6]. Frequent duplications are probably responsible for expansion of the RXLR family, and newly duplicated genes are rapidly dispersed to other loci in the genome [16].

Oomycete pathogens also secrete another large repertoire of cytoplasmic effectors, termed Crinkler (CRN). CRN was first identified from *P. infestans* as a protein that triggered leaf-crinkling and cell-death phenotype in plants [18]. The family of CRN effectors shows extensive expansion in all sequenced *Phytophthora* species [6], [10], [19]. CRN effectors are also found in *Batrachochytrium dendrobatidis*, an amphibian pathogen, but this is likely due to a horizontal transfer event from oomycetes as CRN effectors are not present in other sequenced fungi or bacteria [20]. Analogous to RXLR effector proteins, the N-terminal region of CRN proteins contains a conserved FLAK motif required for CRN effector targeting and translocation [21]. The C-terminal region is diverse in structure and is required to control virulence [6]. CRN1 and CRN2 of *P. infestans* were previously observed to elicit plant cell death [18]. The other two CRN effectors identified from *P. sojae* have opposing roles in pathogenesis; PsCRN63 induces PCD while PsCRN115 blocks it [22]. Despite the fact that several evidences of recombination events were observed to drive CRN diversity in *P. infestans* [6], little is known regarding the evolution and function of the CRN family.

Thus, we combined bioinformatics and experimental approaches to investigate the evolution and function of CRN effectors in *P. sojae*. The results suggest that many CRN effectors are specifically expanded in *P. sojae* and undergo a birth-and-death pattern. Both fragment recombination and gene duplication contribute to the expansion of CRN effectors in *P. sojae*. Surprisingly, we demonstrated that the majority of CRN effectors could suppress PCD induced by distinct elicitors (PAMP and other effectors) while few trigger it. These results illustrate the mechanism of expansion and neofunctionalization of the CRN gene family in *P. sojae*, increasing our understanding of the coevolution between oomycete pathogens and their hosts.

## Materials and Methods

### Data Sets and Sequence Analysis

The genome sequences of *P. sojae* and *P. ramorum* were downloaded from Joint Genome Institute (http://genome.jgi-psf.org), while the *P. infestans* genome was acquired from the Broad Institute (http://www.broadinstitute.org). All known CRN sequences in the three *Phytophthora* species were retrieved from previous reports [6], [10] and renamed by the order of localization on different scaffolds [10]. The RXLR sequences of *P. sojae* and *P. ramorum* were reported by Jiang et al [16]. CRN pseudogenes were adjusted by removal of one or more stop codons or by correcting frame shifts via deleting one or two nucleotides. The sequences were aligned by performing the ClustalX program [23], using default settings, followed by manual inspection of the complete alignment. To investigate the evolutionary relationships of CRN effectors among three *Phytophthora* species, we created orthologous gene groups (OGGs) using the following methods. First, an all-versus-all blastp analysis was performed using an E-value of 1e-5 as cut off value. Next, this blast score was used as input for the MCL Markov clustering program [24], using an inflation parameter of 6. Each resulting cluster is considered an OGG. Sequence divergence is defined as one minus sequence identity. The identity of the most similar paralog of any CRN protein or RXLR protein in *P. sojae* was calculated using the computation of pairwise distances with Poisson model and 1000 bootstrap replicates implemented in Molecular Evolutionary Genetic Analysis (MEGA) version 4.1 [25]. Within the same method, PsCRN (PsRXLR) and PrCRN (PrRXLR) were compared against *P. ramorum* and *P. sojae* proteins, respectively, to get the identity of the most similar ortholog between two *Phytophthora* species. To calculate the average expression value for CRN genes, pseudogenes were excluded, and each functional gene was searched against 21-bp (CATG+17 bp) DGE tags generated by Ye et al [26] and redundant tags were removed. Some CRN genes mapped to the same tag because of high sequence similarity, so only the average expression values were calculated through dividing the sum of transcript copies per million tags (TPM) for all mapped tags by the number of calculated genes. For comparison, the average expression values of 385 PsRXLR genes [16] and 8 Alpha-tubulin genes of *P. sojae* were also calculated within the same method. Alpha-tubulin genes were designated as housekeeping genes.

### Estimation of the Numbers of Genes in the Ancestral Species and those of Gene Gains and Losses

To estimate the numbers of CRN genes in the ancestral species and gains and losses of genes during evolution, the modified reconciled-tree method was performed by comparing the bootstrap condensed gene tree with the species tree under the parsimony principle [27]. The species tree of three *Phytophthora* species was previously reported [28], [29]. Because of the large number of CRN sequences, the phylogenetic tree of 714 CRN genes is not well resolved. So we estimated the gene gain and loss events of each OGG separately. For each OGG, the Neighbor-Joining tree was constructed using MEGA [25] program with the Poisson correction distances and 1000 bootstrap replicates. We then combined the results from all OGGs to obtain the final numbers of gene gains and losses.

### Analysis of Gene Duplication

Duplication events among *P. sojae* CRN effectors were identified using the MEGA program. A sequence similarity of over 90% was considered sufficient to identify duplicated genes. All duplicated genes were classified into different categories by examining their genomic locations. If the two CRN genes of a pair were near one another, they were labeled as tandem duplications. If the duplications belonged to two copied blocks of genes at unlinked sites, they were labeled as segmental duplications. The remaining pairs were labeled singleton duplications, because the evidence suggested that a single gene was copied to another, unlinked, locus. Repetitive sequences around the duplicated genes were identified by searching the Repbase repeat library with the program CRNSOR (www.girinst.org/censor/index.php) [30].

### Detection of Recombination Events

Evidences for recombination events were sought with the Recombination Detection Program (RDP3) [31] with the default values. Several specific methods including RDP, GENECONV, BOOTSCAN, and 3SEQ implemented in the RDP3 program were used with default settings. Fragment recombination is an ectopic recombination that fragment sequence exchange between different CRN gene loci, thereby unrelated CRN genes share conserved fragments. To detect the conserved fragments between different OGGs, *P. sojae* CRN genes were searched against each other using Blastn program, and blast hits meeting the threshold (E value cutoff: 1E-5, hit length ≥40 bp, hit identity >95%, and hit occurred in at least two different OGGs) were defined as conserved fragments. The same blast parameters were used when looking for conserved fragments within the same OGG.

### Detection of Selective Pressure

To detect selective pressure acting on CRN genes, the ratio between nonsynonymous substitution and the synonymous substitutions was measured on protein coding genes. The ω <1, ω = 1, and ω >1 represent negative selection, neutral selection, and positive selection, respectively. To test the selective pressure acting on the entire ORF sequences, YN00 program implemented in the PAML package [32], [33] was performed. In this analysis, we did not use the partial CRN genes because most of them were much shorter than intact genes and would influence results. To detect the particular amino acid sites under positive selection, we applied two pairs of site models: M1a/M2a and M7/M8 in the CODEML program implemented in the PAML package. The null models (M1a and M7) use distributions that do not allow for sites at which ω >1, whereas the alternative models (M2a and M8) do. The likelihood ratio test statistic between the null model and the alternative model was compared with the χ^2^ distribution with two degrees of freedom. The Bayes empirical Bayes method was employed to identify amino acids sites potentially evolving by positive selection [34].

### Plasmid Construction and *Agrobacterium Tumefaciens* Infiltration Assay

pGR107 constructs containing PsCRN63, PsCRN115, PsojNIP, INF1, Avr1k, GFP, Avh241, the combination of Avr3a and R3a were described previously [22]. The oligonucleotides used for the other plasmid constructions are documented in Table S6. Then, the amplicons were cloned using appropriate restriction enzymes into the PVX vector pGR107 [35]. *Nicotiana benthamiana* were maintained under greenhouse conditions with a temperature of 22–25°C and high light intensity throughout the experiments. Multiple independent experiments using agro-infiltration assays with recombinant *Agrobacterium tumefaciens* GV3101 (carrying each plasmid) were conducted as previously described [22]. For cell death induction experiments, *A. tumefaciens* cell solutions carrying the respective constructs were infiltrated into *N. benthamiana* leaves with final OD_600_ of 0.4–0.6. For cell death suppression experiments, *A. tumefaciens* cells expressing the CRN effectors (pGR107 constructs described earlier) or controls were first infiltrated with a final OD_600_ of 0.5. The infiltration sites were challenged 12h later with recombinant *A. tumefaciens* carrying the cell death inducing genes (PsCRN63, PsojNIP, INF1, Avh241 and the combination of Avr3a and R3a) at a final OD_600_ of 0.3–0.6 [22].

## Results

### CRN Effectors Species-specifically Expanded in *Phytophthora* Species

The CRN family is widely expanded in *Phytophthora* species, especially *P. infestans* (196 genes and 255 pseudogenes), *P. sojae* (100 and 102, respectively) and *P. ramorum* (19 and 42, respectively) [6], [10]. To identify the extent to which the CRN genes have orthologs among the three *Phytophthora* species, CRN pseudogenes were adjusted first, and then all 714 CRN proteins were clustered into orthologous gene groups (OGGs) by use of the MCL Markov clustering program with pairwise blastp protein similarities and an inflation value of 6. We identified 45 OGGs containing 2 to 96 genes (Table S1). A total of 608 CRN genes were widely distributed in 20 OGGs,each of which included more than 10 genes. The distribution numbers in most OGGs were dramatically different among three species (Table 1 and Table S1). In the largest group OGG1, the number in *P. infestans* was five times bigger than that in *P. sojae* or *P. ramorum*. *P. sojae* exhibited the most abundant CRN genes in OGG4, and the phylogenetic tree of this group revealed that all the CRN genes from *P. ramorum* and *P. infestans* were clustered with part of CRN genes from *P. sojae* and were mainly distributed in two clades (Fig. S1). The left clades only contained CRN genes from *P. sojae*, indicating specific expansion in *P. sojae*. Furthermore, 37 CRN genes which clustered into 12 OGGs were distributed only in one of three *Phytophthora* species, and only 5 OGGs contained two or more genes in all species (Table S1).

**Table 1:** Number of CRN genes in each OGG.

Organism^a^	OGG1	OGG2	OGG3	OGG4	OGG5	OGG6	OGG7	OGG8	OGG9	OGG10
*P. sojae*	14	6	6	50	10	4	4	9	11	1
*P. infestans*	71	64	65	7	3	27	23	16	14	23
*P. ramorum*	11	7	1	7	1	1	3	2	1	0

a, The numbers of CRN genes in ten orthologous gene groups (OGGs) are included as an example. All OGGs are shown in Table S1.

**Figure 1:**
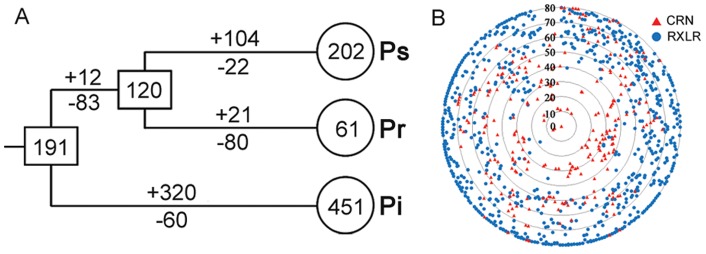
Comparative analysis of the CRN family in different *Phytophthora* species. (A) Estimation of gene number in ancestral species and gains and losses of genes during CRN evolution in three *Phytophthora* species. The numbers in the circles and rectangles represent the number of genes in extant and ancestral species, respectively. The numbers with plus and minus signs indicate the numbers of genes gained and lost, respectively. Ps, Pr and Pi represent *P. sojae*, *P. ramorum* and *P. infestans*, respectively. (B) Sequence divergence of CRN proteins between *P. sojae* and *P. ramorum*. Each triangle or circle represents one sequence identity. Each radius ranges from 0 (center) to 80 (outer circle) representing 100–20% (or less) identity. The distribution along each circumference is random. CRN effectors are marked with red triangles and RXLR proteins are marked with blue circles.

To explore the evolutionary changes of CRN genes in *Phytophthora*, we estimated the number of CRN genes in the most recent common ancestor (MRCA) and gene gains and losses during the evolution of three *Phytophthora* species using the modified reconciled-tree method [27]. This method is based on comparison of a bootstrap condensed gene tree with the species tree, and the numbers are estimated based on the parsimony principle. The gene gain and loss events were estimated for each OGG separately, and then summed up to get the total number. As shown in Figure 1A, there were 191 ancestral CRN genes in the MRCA of *Phytophthora*, and 320 gene gains and 60 gene losses in *P. infestans* since its divergence, suggesting that the gain events outnumbered the loss events. Then the number of CRN genes was reduced to 120 with 83 genes lost in the MRCA of *P. sojae* and *P. ramorum*. *P. sojae* and *P. ramorum* gained 104 and 21, and lost 22 and 80 genes, respectively, since their divergence. Clearly, the numbers of genes gained in *P. infestans* and *P. sojae* were much greater than those lost; however, the opposite was observed in *P. ramorum*. The results indicate that most CRN genes were expanded in a species-specific manner after species divergence.

### CRN Effectors are Moderately Conserved between *P. sojae* and *P. ramorum*

We performed comparative analyses of CRN genes between two related species, *P. sojae* and *P. ramorum* by calculating pairwise sequence similarity [16]. The average sequence identity of the most similar orthologs of any CRN protein in another species was 52% (Fig. 1B). In contrast, the average identity of RXLR effectors was only 31% [16]. 92% of the RXLR superfamily showed sequence divergence of >50% (Fig. 1B) [16]. However, only about half of the CRN family showed sequence divergence of >50% (Fig. 1B), indicating that CRN genes were more conserved than the RXLR genes.

**Figure 2:**
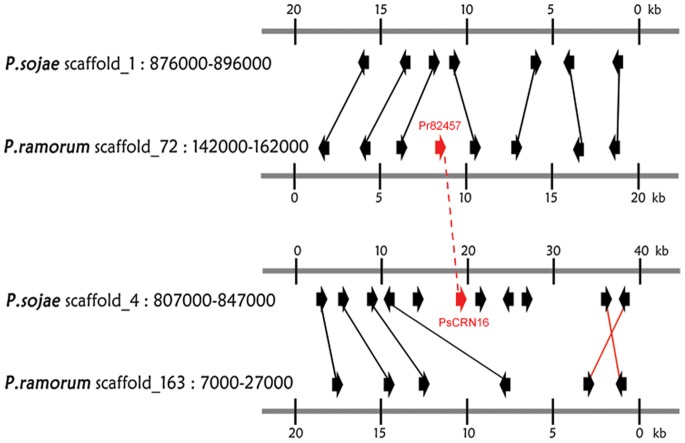
CRN effectors disrupt regions of collinear gene order. Filled red arrows represent two orthologous CRN genes. Collinear orthologous gene pairs are connected by black (direct) or red (inverted) lines.

In the *P. sojae* genome, 202 CRN genes were distributed widely on 118 scaffolds. Two consecutive CRN genes were grouped as a cluster if the distance between them was <100 kb, regardless of the presence of other genes within the cluster. Using this definition, we identified 43 CRN clusters accounting for 102 (50.5%) CRN genes (Table S2). The number of CRN genes per cluster ranged from 2 to 4, with an average of 2.3, and most CRN genes in the same cluster typically originated from diverse OGGs (Fig. S2). The largest CRN cluster spanned one contig of 11 kb on scaffold 4, and 4 CRN genes belonging to 3 OGGs were found in this contig. Another case is that 3 CRN genes from 3 OGGs were located in one cluster on scaffold 14. There was extensive colinearity of orthologs between *P. sojae* and *P. ramorum* genomes [10]. A comparative analysis of the locations of CRN genes between the two genomes was performed, and the majority (78%) were located in regions showing synteny (Table S2). Extensive local rearrangements were discovered at the conserved locations of these CRN genes. For example, one pair of CRN orthologs, PsCRN16 and Pr82457, shared 78% sequence similarity, but deletion events occurred at both colinearity (Fig. 2). These results suggest that gene orientation and local genome rearrangements are numerous.

### CRN Effectors in *P. sojae* could be Grouped into Two Classes

**Figure 3:**
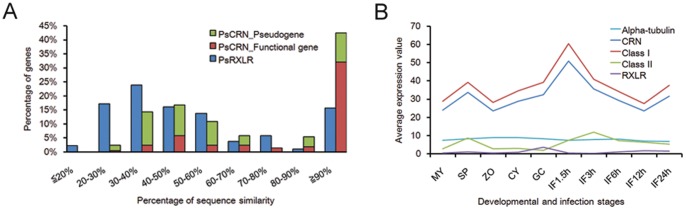
Comparative analysis between Class I and Class II CRN effectors. (A) Sequence divergence of CRN effectors compared to their closest paralogs in *P. sojae*. RXLR effectors were analyzed for comparison. (B) Average gene expression levels of the entire CRN family, Class I, Class II, Alpha-tubulin and RXLR family of *P. sojae* at ten sampled stages: mycelia (MY), zoosporangia (SP), zoospores (ZO), cysts (CY), germinating cysts (GC), and IF1.5h to IF24h (samples from 1.5 to 24 h after infection of soybean leaves, respectively). Only the average expression values of functional genes were calculated.

Of the 202 CRN effectors in the *P. sojae* genome [10], 134 (66.3%) CRN proteins shared >50% identity with their closest paralogs, compared to only 157 of 385 (40.8%) in the RXLR family (Fig. 3A). CRN effectors sharing >50% sequence identity were designated as Class I (family proteins). The remaining CRN effectors showed less similarity and were called Class II (orphan proteins). A total of 82 and 18 functional genes and 52 and 50 pseudogenes were found in Class I and Class II (Table S2), respectively. The average gene-expression patterns of functional genes were calculated based on transcriptional profiling of *P. sojae* at ten different developmental and infection stages [26]. The average expression levels of CRN genes were considerably higher than those of the RXLR genes and Alpha-tubulin genes, indicating that CRN effectors may play important roles in pathogenicity. The average expression levels of Class I genes were significantly upregulated during early infection. In contrast, the gene expression levels of Class II genes were much lower (Fig. 3B).

### Gene Duplication Contributes to CRN Family Expansion

We considered that two CRN genes were duplicated from a common ancestral gene only if they had >90% sequence identity at the DNA level. Altogether, 93 of 202 (46.0%) CRN genes with a paralog had >90% identity to their closest paralogs, most likely the result of recent duplication events. The 93 duplicated CRN genes were divided into 16 duplicated gene groups (DGGs) (Table S2) and genes in the same DGG were considered to be duplicated from a common ancestral gene. The sequences flanking these duplicated genes were examined to gain additional information on their physical relationships. Almost all of the duplicated genes had identical sequences in upstream or downstream regions, but with different sequence lengths. For example, PsCRN44, PsCRN57, and PsCRN105 underwent gene duplication events and had 0.2 kb of almost identical sequence upstream of the start codon, however the extended similar 0.2 kb region was present only between PsCRN44 and PsCRN57. More obvious, a similar segment of 5.6 kb was present downstream of all the above three CRN genes, while an extended 11.5 kb identical segment was shared only between PsCRN44 and PsCRN57. It was possible that the upstream and downstream flanking sequences of PsCRN105 had been altered extensively by sequence rearrangement.

**Figure 4:**
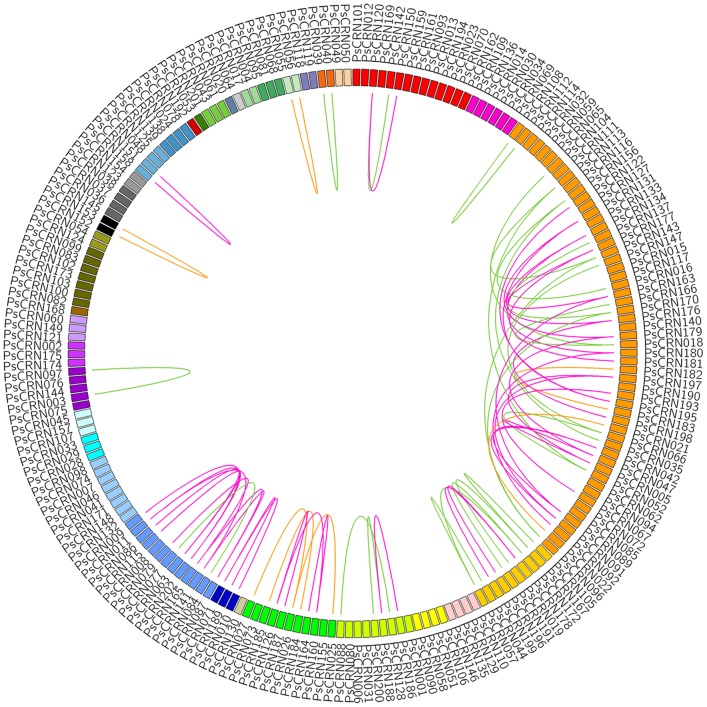
Three duplication categories occurred in CRN family. Three different duplication categories were indicated by differently colored lines connecting duplicated pairs. All the CRN genes were arranged by the OGG sequence, and each OGG was showed with a color. The purple line represents singleton duplication, and green line indicates segmental duplication, while orange line represents tandem duplication.

To trace the duplication history of the CRN repertoire, we investigated the distribution of these duplicated genes across the whole genome and found three duplication categories, including tandem duplication, segmental duplication, and singleton duplication (Fig. 4). In summary, 13 CRN genes experienced 6 tandem duplication events, where the duplicated gene was contiguous with the original duplication, such as the duplication that yielded PsCRN55-PsCRN56 over 1.6 kb on scaffold 21. Another 35 CRN genes were thought to undergo segmental duplication events, leading to duplication of a genomic segment to unlinked scaffolds, even when a segment was translocated to the same scaffold. The segment may be composed of two or more genes. For example, a cluster on scaffold 5 including two adjacent but unrelated CRN genes, PsCRN18 and PsCRN19, was identical to another cluster on scaffold 159 containing PsCRN166 and PsCRN167. Over 90% sequence similarity was detected between PsCRN18 and PsCRN166, as well as between PsCRN19 and PsCRN167. Besides the above two duplication categories, singleton duplication events occurred more frequently in the CRN family, accounting for 45 genes. Singleton duplication involved the duplication of individual gene to an unlinked locus. For example, a single PsCRN196 was similar to PsCRN199 on another scaffold with highly diverse genes in the upstream or downstream regions. We detected 2kb flanking sequences located upstream and downstream of the duplicated genes using the CRNSOR repeat masking software and predicted 283 repetitive elements in 72 (77.4%) CRN genes (Table S3). The most abundant repetitive element is Polinton DNA transposon, which was present in the flanking sequences of 63 duplicated CRN genes. In addition, 30 Gypsy and 24 Copia LTR retrotransposons were discovered, respectively. Hence, transposable elements could have driven gene duplications by inadvertently carrying copies of genes during transposition events and/or by facilitating unequal crossovers.

To determine which type of selective pressure (positive, neutral, or purifying selection) shaped the evolution of these genes after gene duplication, we assessed selective pressure of the duplicated CRN genes of each DGG using the ratio (ω = *d*_N/_ *d*_S_) of the nonsynonymous (*d*_N_) substitution rate to the synonymous (*d*_S_) substitution rate at the codon level [32,32]. First, we calculated *d*_N_ and *d*_S_ values across the entire ORF sequences using YN00 program. The *d*_N_ value was greater than *d*_S_ (ω = *d*_N_/*d*_S_ >1) in at least one pairwise comparison in 8 of 16 DGGs (Table 2). In the large DGGs (more than three members), positive selection was more evident. Meanwhile, positive selection typically acts on particular amino acid sites within a given protein. To detect the particular amino acid sites under positive selection, we applied two pairs of site models, namely, M1a/M2a and M7/M8 implemented in the CODEML program of the PAML package. Of the 9 DGGs containing more than three members, 7 DGGs showed statistically significant evidence of positive selection (p<0.05; M1a and/or M7 rejected in favor of M2a and/or M8). The Bayes empirical Bayes method implemented in PAML identified more than 10 codon sites in each DGG showing evidence of positive selection (ω >1; posterior probability >95%; Table 2). These results suggest that positive selection acted on the duplicated CRN genes.

**Table 2:** DGGs under positive selection.

DGG	Number^a^	*d*_N_/*d*_S_ among Pairwise Comparisons				Identification of Positively Selected Sites	
		Number Analyzed^b^	Highest *d*_N_/*d*_S_^c^	*d*_N_	*d*_S_	Significance^d^	Number of PSS^e^
DGG1	7	4	0.96	0.1004	0.1046	Yes	34
DGG2	5	5	1.006	0.0370	0.0368	Yes	21
DGG3	9	7	1.19	0.0989	0.0831	Yes	45
DGG4	10	2	3.64	0.0207	0.0057	Yes	19
DGG5	24	12	1.58	0.0207	0.0131	Yes	14
DGG6	10	8	1.30	0.0364	0.0281	Yes	21
DGG7	5	3	0.88	0.0602	0.0686	Yes	10
DGG8	6	6	1.05	0.3541	0.3372	No	-
DGG9	3	2	0.54	0.0245	0.0452	No	-
DGG10	2	2	∞	0.0070	0		
DGG11	2	2	NA	0	0		
DGG12	2	2	1.008	0.0239	0.0237		
DGG13	2	2	0.69	0.0330	0.0481		
DGG14	2	2	0.68	0.0450	0.0663		
DGG15	2	2	0.71	0.0270	0.0379		
DGG16	2	2	0.76	0.1414	0.1852		

a, Numbers of sequences in the listed DGGs.

b, Numbers of sequences analyzed in pairwise comparisons. Partial sequences were excluded.

c, Highest dN/dS ratio among all pairwise comparisons within one PGG. ∞ refers to pairwise comparisons in which dN>0 and dS = 0. NA means two sequences are the same in one pair.

d, Yes indicates sufficient evidence for positive selection if the two likelihood tests (M2a vs. M1a, M8 vs M7) performed show statistical significance.

e, Number of PSS identified with posterior probability P>95%.

### Recombination Contributes to Sequence Divergence

**Figure 5:**
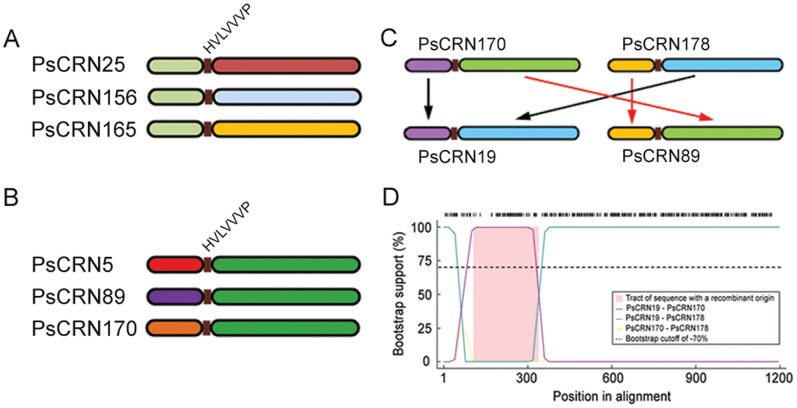
Recombination patterns contribute to sequence divergence. (A) CRN genes with different C-terminal regions were shown to have nearly identical N-terminal sequences. (B) CRN genes with different N-terminal regions were shown to have nearly identical C-terminal sequences. (C) CRN genes were generated by combining the N- and C-terminal sequences of the other two CRN genes. (D) Bootscanning plot of PsCRN19 gene sequence against PsCRN170 and PsCRN178 is depicted, with region from nucleotides 44–341 highlighted. Plot is modeled on a pairwise alignment with a step size of 20 and 1000 bootstrap replicates.

CRN effectors exhibited evidence of recombination in *P. infestans*, and the conserved HVLVVVP motif was found to be the recombination breakpoint [6]. To investigate the recombination events that occur between CRN genes in *P. sojae*, the CRN sequence was divided into the N-terminus and C-terminus using HVLVVVP motif as the demarcation, and we performed numerous recombination detection methods implemented in the RDP3 program [31]. Evidences of recombination events driving CRN diversity were identified in three different patterns (Fig. 5, Fig. S3). First, CRN genes with highly similar N-terminal sequences had unrelated C-terminal regions, accounting for 32% of the CRN genes; these included PsCRN25, PsCRN156, and PsCRN165 as shown in Figure 5A. The second recombination pattern was that CRN genes with different N-terminal regions had nearly identical C-terminal sequences. A total of 35% of CRN genes experienced this recombination event including PsCRN5, PsCRN89, and PsCRN170 as shown in Figure 5B. Furthermore, a novel CRN gene can be generated by combining the N-terminal (C-terminal) region of one CRN gene and the C-terminal (N-terminal) region of another CRN gene. Lots of CRN genes experienced this type of intergenic crossover including PsCRN19, PsCRN170, and PsCRN178 (Fig. 5C). PsCRN19 was inferred to be a recombinant of PsCRN170 and PsCRN178. Statistical support for this event was high and provided by multiple algorithms (RDP, P = 3.9*10–50; GENECONV, P = 4.8*10–55; BootScan, P = 8.3*10–54; and 3Seq, P = 3.1*10–59) within the RDP3 package, and was obvious based on visual inspection (Fig. 5D).

### Fragment Recombination Occurs in *P. sojae* CRN Effectors

**Figure 6:**
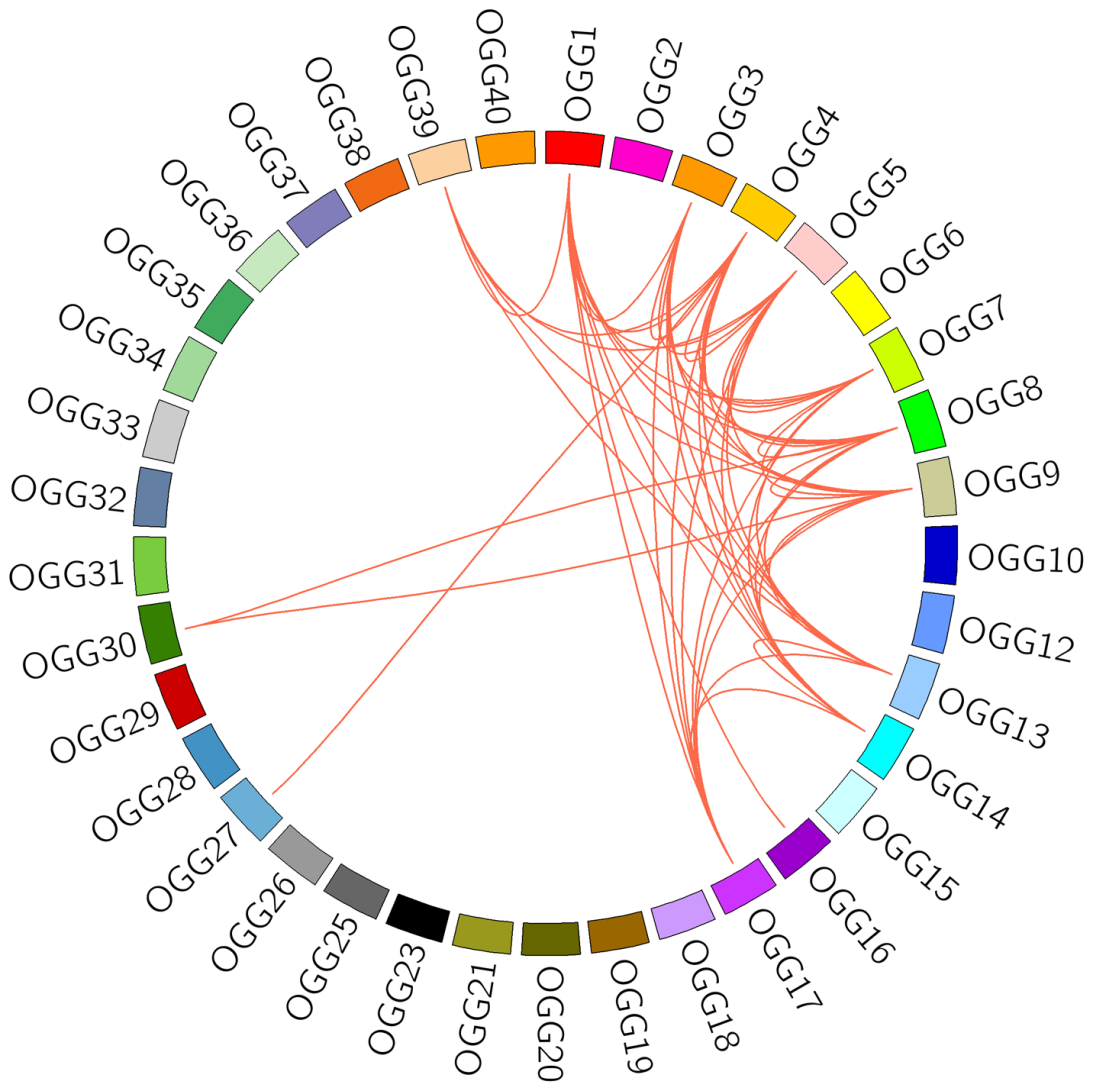
Frequent fragment recombination among different OGGs. Any two OGGs sharing the conserved fragment were connected with a red line.

Although we identified recombination events involving exchanges of large fragments, some small fragment recombination events were also investigated. For example, PsCRN91 and PsCRN172 shared little similarity across the entire ORF, excluding a conserved fragment of 250 bp in the N-terminal region (Text S1), which likely underwent fragment exchange. To identify all fragment recombination events, we examined the conserved fragments in at least two OGGs, according to the similar sequence search described in methods. A total of 78 conserved fragments were identified with lengths ranging from 40 to 303 bp (Table S4), suggesting that fragment recombination events were frequent among different OGGs (Fig. 6). Moreover, we investigated fragment recombination among genes within the same OGG. Frequent fragment exchanges were discovered in the same OGG, especially in a large group OGG4, involving 40 conserved fragments (Table S5). The above results indicate that fragment recombination can result in a complex network among CRN effectors. In addition, most of the fragment recombination events were restricted to genes found in duplicated regions of the genome, indicating that the sequence exchange occurred prior to the duplication of genomic regions.

### *P. sojae* CRN Effectors Suppress Diverse PCD Inducers

To determine whether diverse *P. sojae* CRNs perturb host cellular processes, we chose OGG5 and 15 randomly selected CRNs (Table S6) from *P. sojae* to test the activity using *Agrobacterium tumefaciens* cells to deliver potato virus X (PVX) vector carrying CRNs. These methods have been widely used for transient expression of genes in *N. benthamiana* and to determine whether particular genes induce or suppress PCD [6], [14], [22].

OGG5 contains 10 genes, and 7 were amplified, excluding 1 pseudogene and 2 C-terminally truncated genes. Surprisingly, 2 of them were present in two copies. Of the 9 amplified genes, 7 genes were thought to be duplicated from a single ancestral gene and have subsequently underwent recombination with 2 other genes (PsCRN167 and PsCRN178) which share the same sequence. Then we selected eight genes for functional analysis. Several known elicitors of PCD were used as positive controls: PsojNIP [36], INF1 [37], PsCRN63 [22], a combination of *P. infestans* Avr3a and potato R3a [38], and Avh241 [39]. We found that only PsCRN172–2, a newly identified copy of PsCRN172, triggered PCD in *N. benthamiana*, while the other seven CRN effectors did not (Fig. S4). Next, we examined whether these CRNs suppressed PCD triggered by other elicitors, and found that all of the CRN effectors in OGG5 suppressed PCD triggered by PsojNIP and PsCRN63, 5 suppressed PCD mediated by Avr3a-R3a protein interaction, 3 in OGG5 blocked Avh241-triggered plant PCD, and 4 inhibited PCD induced by INF1. PsCRN172–1 and PsCRN172–2, together with PsCRN63 and PsCRN115 [22], had opposing necrosis-inducing effects, although they have a high sequence similarity, mostly as a result of strong positive selection after gene duplication.

Among the randomly selected 15 CRN effectors (Table S6) from different OGGs, only PsCRN63 had PCD-inducing activity [22]. A total of 10 CRN effectors inhibited PCD triggered by PsCRN63, 7 suppressed PCD mediated by the Avr3a-R3a protein interaction, 6 blocked Avh241-triggered plant PCD, and 13 suppressed PCD triggered by PsojNIP. Moreover, prior infiltration with *A. tumefaciens* cells containing the GFP gene (a negative control) did not protect against PCD. A previously identified PCD suppressor, Avr1k, was used as a positive control and blocked PCD triggered by all of the tested elicitors. Overall, only few CRN effectors could induce PCD while most of others suppress PCD triggered by other inducers.

## Discussion

This study on the evolution and function of CRN family effectors revealed three major findings. First, the CRN family expanded after the divergence of *Phytophthora* species and has evolved in a species-specific manner via a birth-and-death mechanism. Second, nearly 50% of CRN effectors underwent gene duplication followed by positive selection, which contributed to the expansion of the CRN family. Meanwhile, frequent fragment recombination events also occurred, leading to diverse sequence structures. Third, the expanded CRN effectors exhibited diverse functions in pathogenesis such as inducing PCD and suppressing PCD through PAMP-triggered immunity or/and effector-triggered immunity. Thus, we propose that gene duplication and fragment recombination may be two mechanisms that drive the expansion and neofunctionalization of the CRN family in *P. sojae*.

The CRN superfamily extensively expands in oomycete pathogens, particularly *Phytophthora* species. Three sequenced *Phytophthora* genomes encode 61–451 CRN genes (more than half of which are pseudogenes) [6], [10], suggesting that the CRN superfamily evolves rapidly, although it is relatively conserved compared to the RXLR family. We showed that the number of CRN genes changed significantly during *Phytophthora* evolution, and some experienced species-specific expansion. However, the reason of why the number of effectors changed extensively during *Phytophthora* evolution remains unclear. One obvious factor is that the CRN genes are typically found in repeat-rich and gene-sparse regions of the genome *in P. infestans* [6], which provides an ideal environment for rapidly evolving genes. The *P. infestans* genome has a large amount of repeat genomic sequences, resulting in more rapid evolution of effector genes, followed by *P. sojae* and *P. ramorum*. Another important factor might be the requirement for a *Phytophthora* pathogen to adapt to a particular life style. The birth-and-death model is one of the most important mechanisms of multigene family evolution [40], and the CRN family is a good example of this process. In *P. sojae*, most novel CRN genes are formed by gene duplication and/or fragment recombination (birth), while some genes are deleted from the genome or become pseudogenes following deleterious mutations (death). The significantly high birth rates in the CRN family raise the question of why pathogens have recruited so many novel genes during evolution. One hypothesis is that CRN effectors are important for pathogenicity because of their virulence, and rapidly generated novel genes could facilitate evasion of plant recognition and benefit parasite colonization.

Compared to the highly diverse RXLR family, the CRN family members are relatively conserved and may have been acquired through a more recent evolutionary event. We observed two mechanisms (gene duplication and fragment recombination) contributing to the evolution of the CRN family. Recombination plays a disproportionately important role in the evolution of bacterial type III secreted effectors (T3SEs). The evolution of T3SEs is strongly influenced by a non-homologous recombination process, called “terminal reassortment”, which generates novel T3SEs with new virulence functions [41]. This type of recombination also occurs in the CRN family, resulting in diverse sequence structures, which might be responsible for effector activity and function. A large majority of gene duplication events were detected and presumably responsible for the expansion of the CRN family, including tandem duplication, segmental duplication, and singleton duplication. Gene duplication, followed by functional divergence of duplicated genes, is an important evolutionary force driving the emergence of novel gene functions. The fate of duplicated genes can be diverse, with different selective pressures acting on the genes [42]. Typically, there are three outcomes after gene duplication: the duplicated gene may perform a new and adaptively favored function (neofunctionalization), become silenced due to deleterious mutations (nonfunctionalization), or adopt part of the tasks of the ancestral gene (subfunctionalization) [43]. There is often acceleration of the evolution rate following gene duplication. Accelerated rates could initially be driven by positive selection for functional divergence or by relaxation of selective constraints. We detected positive selection in more than 40% of the examined DGGs by calculating the ω values across the entire ORF sequences. Some positively selected sites were also identified using the maximum likelihood method.

The *P. sojae* genome contains a large number of CRN effectors, together with the above two evolutionary mechanisms, raising key questions regarding their shared contributions to functions. In total, 26 CRN effectors, including 15 randomly selected genes from different OGGs and 11 genes in OGG5, were examined to determine whether they could trigger or suppress PCD induced by other elicitors. According to the data, only one CRN gene (PsCRN172–2) triggered PCD while its duplicated copy (PsCRN172–1), which only differed by seven amino acids, did not. This is likely due to neofunctionalization driven by positive selection. Together with the activity of PsCRN63 [22], these results suggest that only a few CRN effectors in *P. sojae* can trigger PCD, which is different from CRN effectors in *P. infestans*. In *P. infestans*, CRN C-terminal regions exhibit a wide variety of domain structures, and many CRN effectors containing diverse C-terminal domains induced PCD [6], suggesting that the C-terminal domains were responsible for the PCD phenotypes. However, both pairs of CRN effectors with conserved C-terminal domains (differing by only a few base changes) exhibited opposing PCD-inducing activities in *P. sojae*, indicating that the diverse C-terminal domains might not be necessary to trigger PCD in *P. sojae*.

Although most *P. sojae* CRN effectors could not induce PCD, they could block PCD triggered by other inducers. The data showed that most of the tested CRN effectors suppressed PCD induced by PsojNIP and PsCRN63, and about half of those could also suppress PCD mediated by Avh241 and Avr3a-R3a protein interaction. The large number of CRN effectors that can suppress PCD involved in PTI and ETI and other host defenses indicates that CRN effectors contribute to virulence by suppressing defensive PCD in plant hosts, which is presumably important during the early biotrophic stage of *P. sojae* infection. The ability of *P. sojae* to suppress or delay the HR of soybean tissue is likely a major component of its pathogenic strategy [15]. Here, we show that CRN effectors may play important roles in *P. sojae* manipulation of the host HR.

To data, six avirulent genes have been cloned from oomycete pathogens, most of which are RXLR family effectors [15]. CRN effectors in *P. sojae* are much more highly expressed than RXLR effectors (Fig. 3B), and few CRN genes that induce HR cause PCD in *N. benthianan* and soybean. However, none of the cloned avirulent genes belongs to the CRN family. One possible reason is that host defense induced by CRN effectors are suppressed during *P. sojae* infection. In the present study, PsCRN63 could be recognized by plants and induce PCD. However, this PCD phenotype could be readily suppressed by most of the tested CRN effectors, suggesting that *P. sojae* contains many secreted CRN effectors, preventing the recognition of other CRN effectors, which is consistent with an effector (ATR39–1) in *Hyaloperonospora arabidopsidis*. ATR39–1 is recognized by *Arabidopsis* ecotype Weiningen and triggers a resistance response when delivered by a surrogate system. However, this recognition does not inhibit the growth of *Hpa* strains expressing ATR39–1, likely *Hpa* contains another secreted effector which prevents the recognition of ATR39–1 [44].

**Figure 7:**
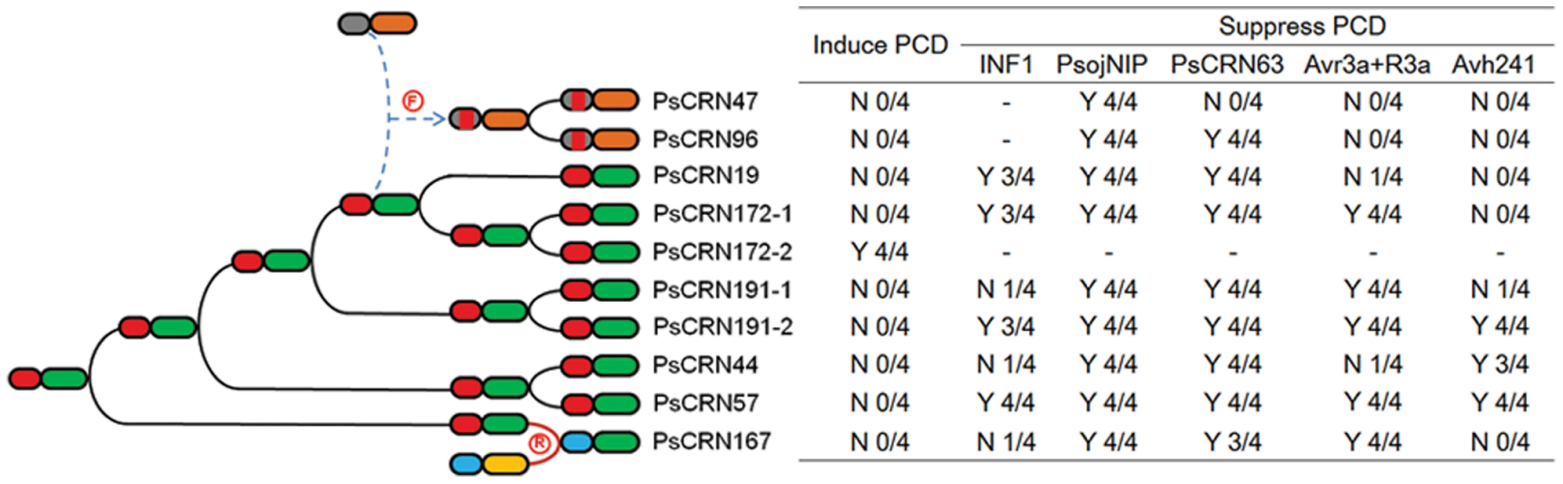
Evolutionary analysis of a cluster of CRN genes in *P. sojae*. The CRN genes in OGG5 are selected to represent the evolutional supposition of CRN family in *P. sojae*. The red letters “F” and “R” in the circles indicate fragment recombination and N-terminal (C-terminal) recombination, respectively. The black line indicates gene duplication. N-terminal and C-terminal structures are represented by different colors. In OGG5, most CRN genes were supposed to be duplicated from one ancestral gene through multiple duplication events. In addition, multiple recombination events occurred frequently, resulting in diverse sequence structures. For example, PsCRN167 was supposed to be the result of N-terminal (C-terminal) recombination, and PsCRN47 and PsCRN96 underwent fragment recombination. Then, accelerated rates were driven by positive selection, causing neofunctionalization, such as PsCRN172-1 and PsCRN172-2. Moreover, subfunctionalization and nonfunctionalization also occurred.

Finally, we select OGG5 as an example and propose a model for the evolution of the CRN family in *P. sojae* (Fig. 7). The arms race between *P. sojae* and plants drives pathogens to improve virulence, resulting in rapid evolution of CRN effectors. CRN effectors evolved rapidly according to the birth-and-death model. Under this model, some new genes were duplicated from one ancestral gene through multiple duplication events. Then, recombination events occurred frequently, resulting in allelic substitution and different sequence structures. There is often acceleration of the evolution rate following gene duplication. Accelerated rates could be driven by positive selection, resulting in neofunctionalization, in which one copy acquires a new function whereas the alternative copy maintains the original function. An alternative more common outcome is that gene duplicates are frequently preserved by subfunctionalization, whereby both copies may become partially compromised and experience degenerative mutations that reduce their joint levels and patterns of activity to that of the single ancestral gene. Some other genes lose function through pseudogene formation following deleterious mutations. The functional diversification helps to destabilize or suppress host cellular processes, thereby compromising host defenses and allowing for successful pathogen colonization and growth.

## Supporting Information

Figure S1 Phylogenetic relationships of CRN effectors in OGG4. The red, green and black circles represent CRN genes from *P. sojae*, *P. ramorum* and *P. infestans*, respectively.
(TIF)

Figure S2 Cluster connections among CRN effectors in *P. sojae*. The CRN effectors in the same cluster are connected by a line. If the cluster only contains two genes, they are connected by a red line. The blue lines connect three genes in the same cluster, which the green lines connect four genes. All the CRN genes were arranged by the OGG sequence, and each OGG was showed with a color.
(TIF)

Figure S3 Recombination connections among CRN effectors in P. sojae. CRN effectors with highly similar N-terminal sequences and unrelated C-terminal regions are connected by blue lines, while purple lines connect CRN genes that share similar C-terminal sequences and diverse N-terminal sequences.
(TIF)

Figure S4 Functional analysis of CRN effectors in OGG5. (A) Phylogenetic relationships of CRN effectors in OGG5. (B) Assay for whether could trigger PCD in *N. benthamiana*. 1, PsCRN167; 2, PsCRN19; 3, PsCRN44; 4, PsCRN191-1; 5, PsCRN172-2; 6, PsCRN172-1; 7, PsCRN191-2; 8, PsCRN57; 9, Avr3a+R3a; 10, PsojNIP; 11, INF1; 12, Avh241; 13, PsCRN63. (C)-(G) Suppression of PCD triggered in *N. benthamiana* by other oomycete elicitors, including INF1, PsojNIP, PsCRN63, Avr3a+R3a, and Avh241.
(TIF)

Table S1 List of the orthologous gene group classification of CRN genes among three *Phytophthora* species.
(XLS)

Table S2 Features of CRN family in *P. sojae*.
(XLS)

Table S3 List of predicted transposable elements located upstream and downstream of the duplicated CRN genes.
(XLS)

Table S4 List of conserved fragments between different OGGs.
(XLS)

Table S5 List of conserved fragments among CRN genes in OGG4.
(XLS)

Table S6 The phenotypes and sequences list of the cloned CRN effectors in *P. sojae*.
(XLS)

Text S1 Nucleic acid sequence alignment of PsCRN120 and PsCRN197.
(DOC)
