# Supplementary figures and images for: Gene Duplication and Fragment Recombination Drive Functional Diversification of a Superfamily of Cytoplasmic Effectors in Phytophthora sojae
Source: PLoS One. 2013 Jul 29;8(7):e70036. doi: 10.1371/journal.pone.0070036 (PMC3726527; doi:10.1371/journal.pone.0070036)

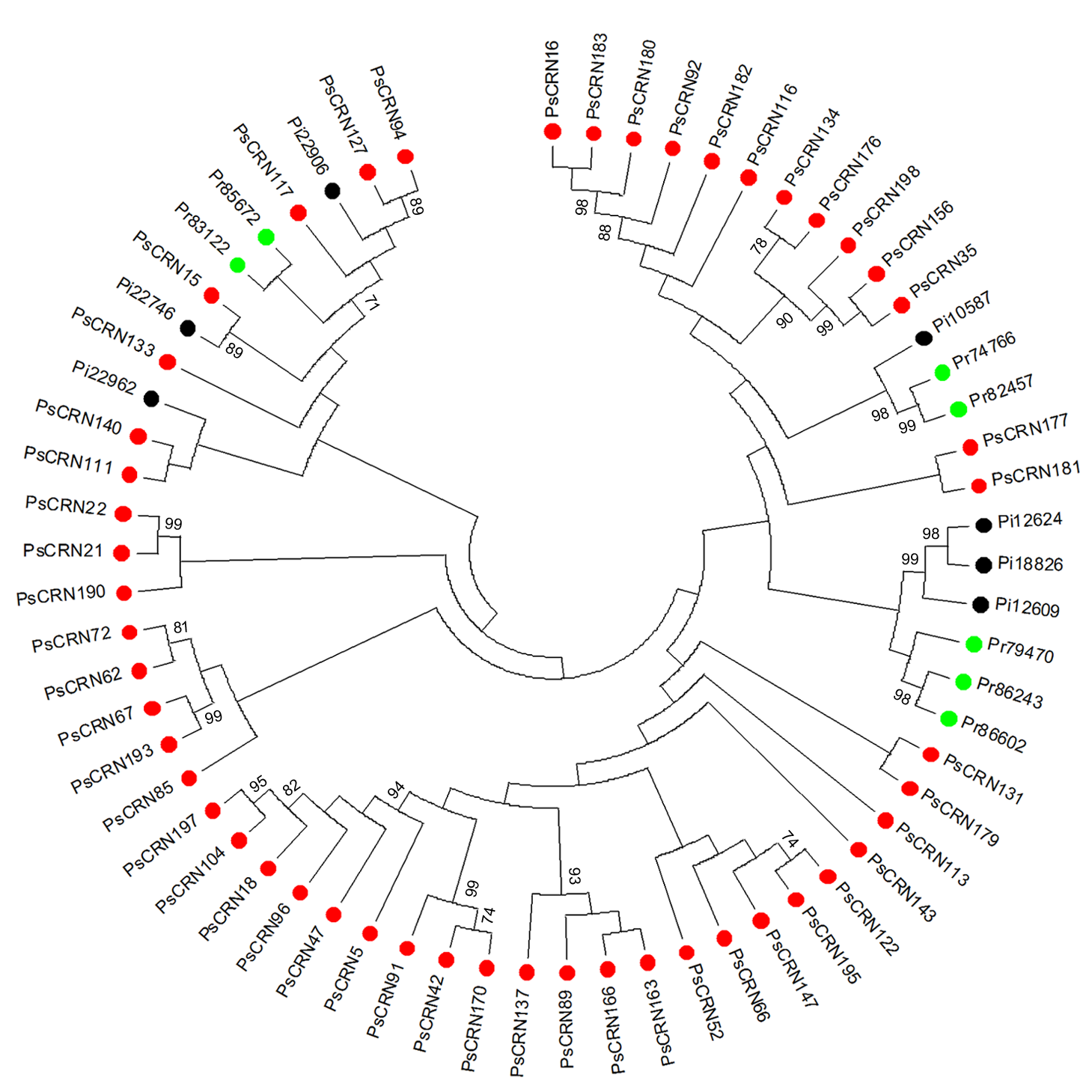

Supplement: Figure S1 — Phylogenetic relationships of CRN effectors in OGG4. The red, green and black circles represent CRN genes from P. sojae, P. ramorum and P. infestans, respectively. (TIF) [file pone.0070036.s001.tif]

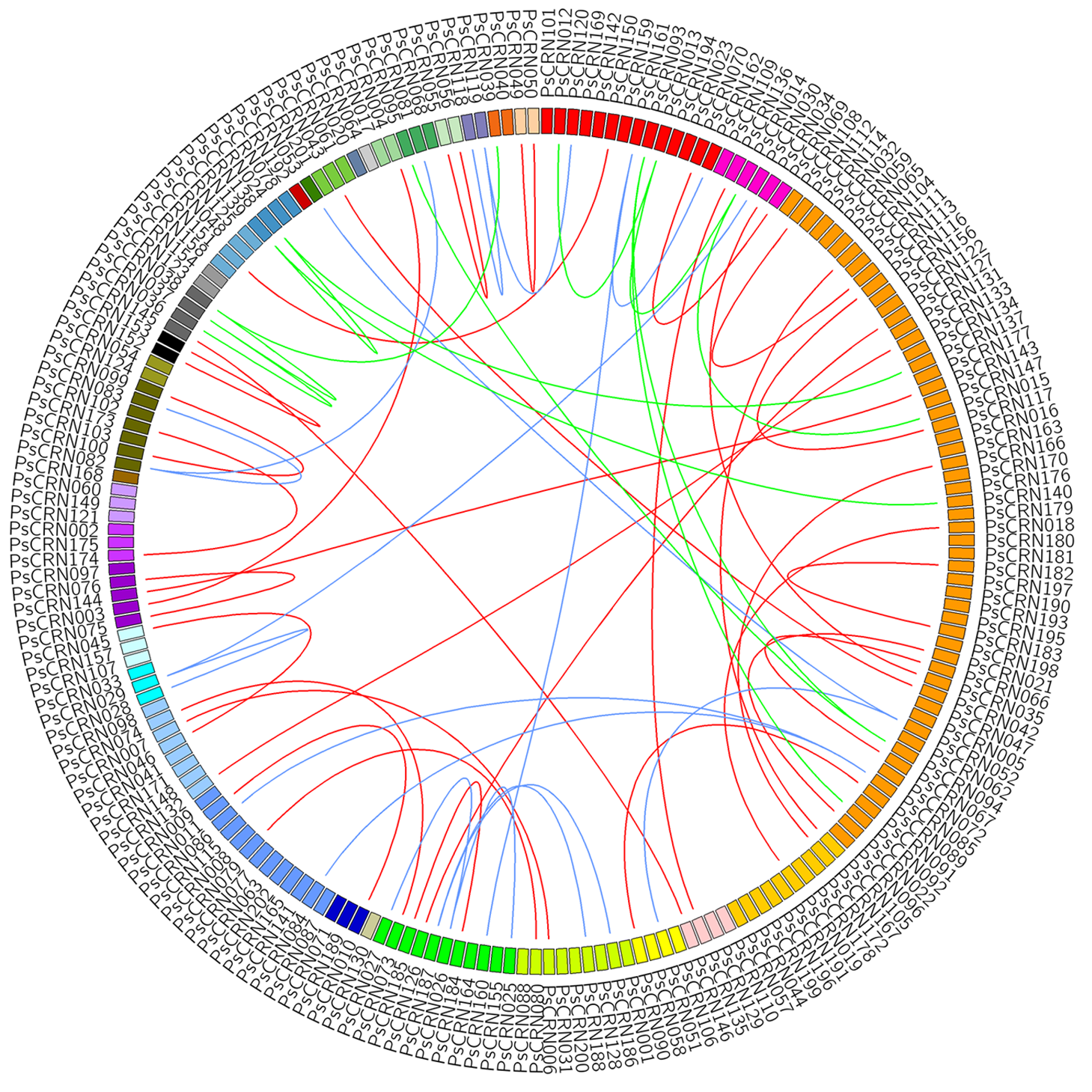

Supplement: Figure S2 — Cluster connections among CRN effectors in P. sojae. The CRN effectors in the same cluster are connected by a line. If the cluster only contains two genes, they are connected by a red line. The blue lines connect three genes in the same cluster, which the green lines connect four genes. All the CRN genes were arranged by the OGG sequence, and each OGG was showed with a color. (TIF) [file pone.0070036.s002.tif]

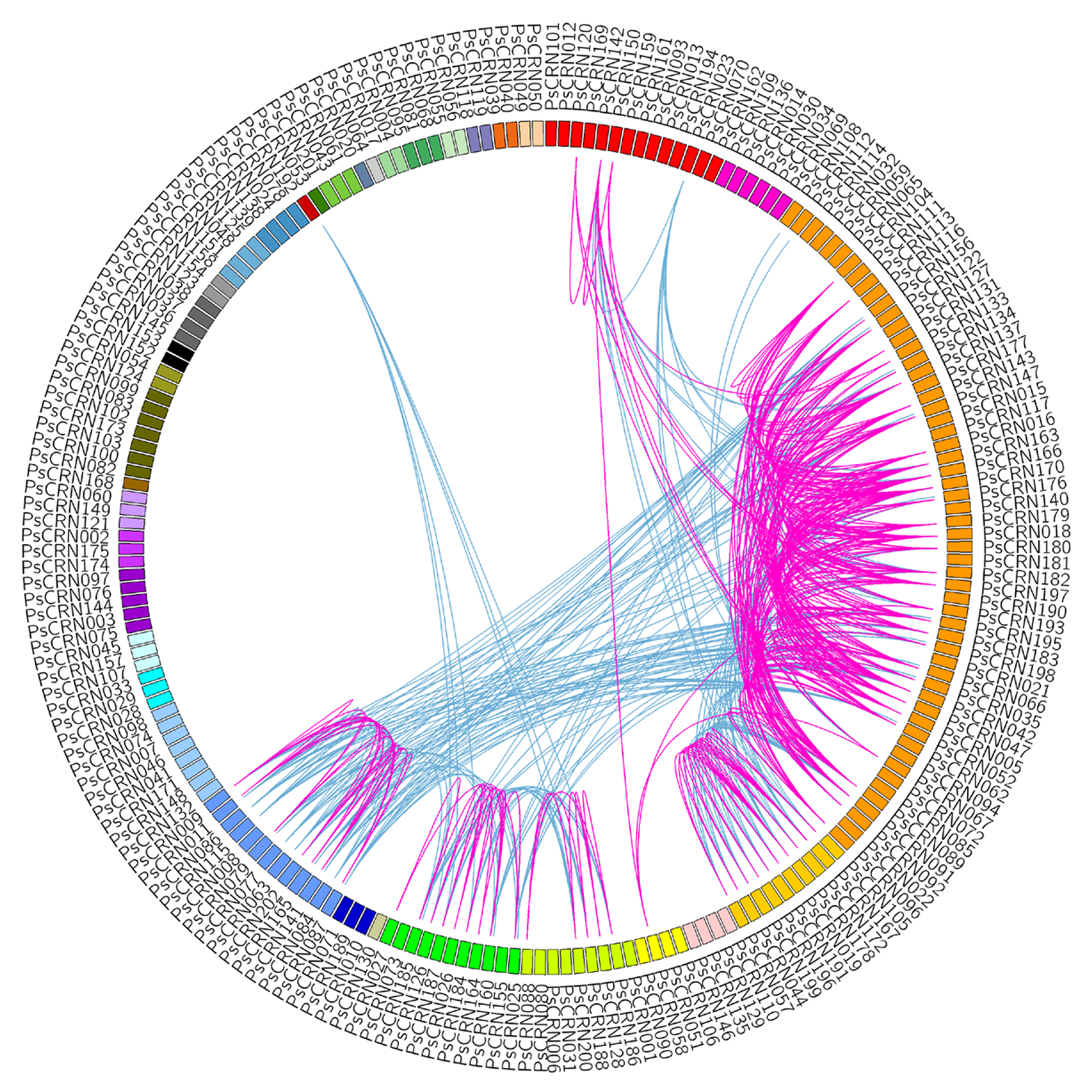

Supplement: Figure S3 — Recombination connections among CRN effectors in P. sojae. CRN effectors with highly similar N-terminal sequences and unrelated C-terminal regions are connected by blue lines, while purple lines connect CRN genes that share similar C-terminal sequences and diverse N-terminal sequences. (TIF) [file pone.0070036.s003.tif]

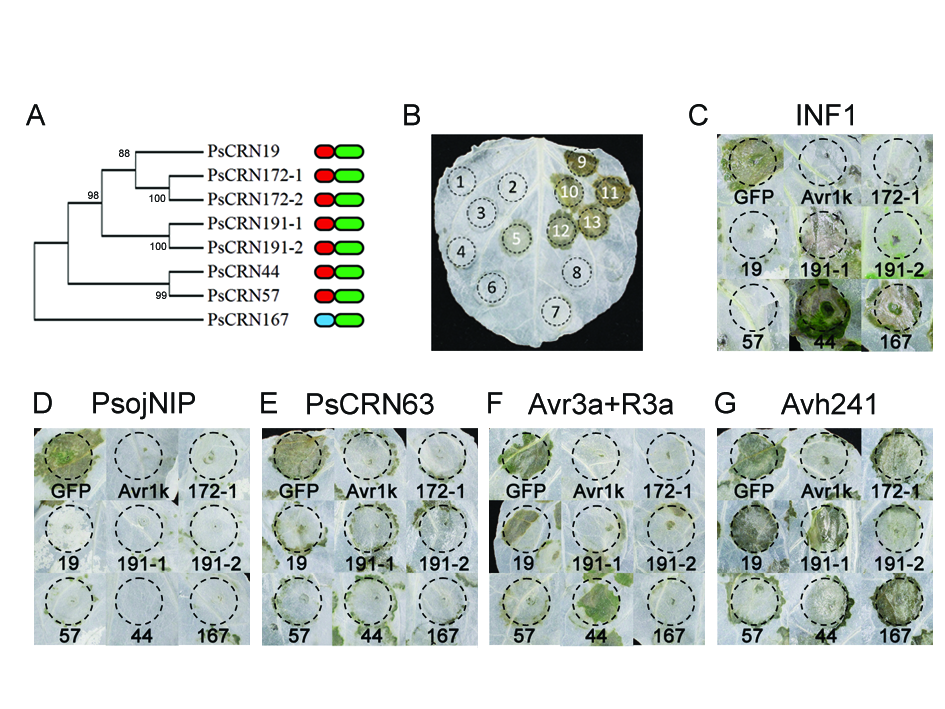

Supplement: Figure S4 — Functional analysis of CRN effectors in OGG5. (A) Phylogenetic relationships of CRN effectors in OGG5. (B) Assay for whether could trigger PCD in N. benthamiana. 1, PsCRN167; 2, PsCRN19; 3, PsCRN44; 4, PsCRN191-1; 5, PsCRN172-2; 6, PsCRN172-1; 7, PsCRN191-2; 8, PsCRN57; 9, Avr3a+R3a; 10, PsojNIP; 11, INF1; 12, Avh241; 13, PsCRN63. (C)-(G) Suppression of PCD triggered in N. benthamiana by other oomycete elicitors, including INF1, PsojNIP, PsCRN63, Avr3a+R3a, and Avh241. (TIF) [file pone.0070036.s004.tif]
