# Supplementary material for: Gene Duplication and Fragment Recombination Drive Functional Diversification of a Superfamily of Cytoplasmic Effectors in Phytophthora sojae
Source: PLoS One. 2013 Jul 29;8(7):e70036. doi: 10.1371/journal.pone.0070036 (PMC3726527; doi:10.1371/journal.pone.0070036)
Supplement: Text S1 — Nucleic acid sequence alignment of PsCRN120 and PsCRN197. (DOC) [file pone.0070036.s011.doc]

Text S1: Alignment of PsCRN91 and PsCRN 172 nucleic acid sequence.

PsCRN172 ATGcTGctGtTgaaCTGcatGcTCGTTGGgGacGgGGGC---GtGaTTtCTaccatCcTT

PsCRN91 ATGgTGaaGcTcttCTGtgcGaTCGTTGGtGcgGcGGGCagtGcGtTTcCTgtggaCaTT

PsCRN172 GAaGaatggaAGaCGGTtgCtctcTTGAAGAAGGCGATCAAGGtGGAGAAGcCGAACGAC

PsCRN91 GAcGcggatcAGtCGGTgtCcgcgTTGAAGAAGGCGATCAAGGcGGAGAAGaCGAACGAC

PsCRN172 TTCAAGGACATCGACGCAGACAAGCTGCAGCTCTTCCTGGCGAAGACGGAGaGCGGCGCG

PsCRN91 TTCAAGGACATCGACGCAGACAAGCTGCAGCTCTTCCTGGCGAAGACGGAGgGCGGCGCG

PsCRN172 TGGCTCTCATCGAAGGACCCTGATaTGATTTCTATGCGAAGTGGAGGCATTCCTGAgCAc

PsCRN91 TGGCTCTCATCGAAGGACCCTGATgTGATTTCTATGCGAAGTGGAGGCATTCCTGAaCAa

PsCRN172 GTGAAGACACTGCTGAACgTGGAAATGGACCCAGCAGAcGAGATTGGCGACGTGTTTGAA

PsCRN91 GTGAAGACACTGCTGAACaTGGAAATGGACCCAGCAGAtGAGATTGGCGACGTGTTTGAA

PsCRN172 GGTGCTCCgACGAAGAAGACGATTCACGTGCTGGTGGTtGcagaTCgGGAGtgccttgAA

PsCRN91 GGTGCTCCaACGAAGAAGACGATTCACGTGCTGGTGGTgGt---TCcGGAGc------AA

PsCRN172 GtccAggACGCtgAAAtTGcAcTGcacCcgaGTCgCaaGAgaCGaTGggacaagttgaAC

PsCRN91 G---AacACGCacAAAcTGgAtTGtggCtt-GTCaCtgGAtcCGtTG-----------AC

PsCRN172 gAgGttCTtgataaGAACAaGAAAGcGAagaagGCTGCcggCTccACgGGtttctcgtac

PsCRN91 aAcGcgCT------GAACAcGAAAGgGAttc--GCTGCaaaCTgtACtGG----------

PsCRN172 gtgtcgttcccggagatTGaCagCaTcAtGC--caGcgACcAaGtTtCgACcatcgtcaa

PsCRN91 ----------------aTGgCgaCgTtAcGCattgGatACtAcGaTcCtAC---------

PsCRN172 aGCctatCcctGaCgAaAAGctTGatGCGcT---GcATagAtAC------------TTTC

PsCRN91 -GCgctgCatcGgCaAcAAGaaTGtcGCGtTctgGtATgaAgACaagaaattgtgtTTTC

PsCRN172 cTaTcTTgaTCaAG----------GCTTtTggAgATatcaTtaCTGgAaaGcgActgcaC

PsCRN91 aTgTtTTatTCgAGacaagtacgtGCTTcT--AtATgctgTcgCTGcAtcGtaAtactgC

PsCRN172 TTCATcG-----TgcCcgTGcTT--------GcAagTGtctgTgcGCTGTTCGAtggagg

PsCRN91 TTCATtGagggcTaaCtcTGtTTcttttataGaAgaTGctgcTttGCTGTTCGAaa----

PsCRN172 cgtccaaatcCTGgCcgAAGaaaccgtGAtTGGGaaacgtgttCACggAgaCgGaGcCTt

PsCRN91 ----------CTGaCttAAG-------GAcTGGG--------cCACaaAcgCtGgG-CTc

PsCRN172 TgaGTTcgtgcttaAGcgcGgcGAGAaaCGaGTTtgcattGtggAaGCgaaacgTgacGA

PsCRN91 TccGTTaactaaccAGgttGttGAGAcgCGtGTTgcaccaGctaAcGCtgtgtcTactGA

PsCRN172 TaTCCAGCaGgGTCTT-------------GcTCaAGcttAcTtGGgAagT----gAAgCA

PsCRN91 TcTCCAGC-GcGTCTTctactgcgactacGtTCcAGacgAtTcGGaAtcTcctcaAAaCA

PsCRN172 CttgcggatgtagaAGgactgcCGAaAgtGTacagcaTtgtcACgAaCtTcTtGgAatgG

PsCRN91 C-------------AGtttcatCGAtAtcGT------TgactACaAgCgTtTcGaAtctG

PsCRN172 GtCttcTC----GAgaAGTcTgGaTg--AaaaaATTGAGcgtGCGAcaccCgTgaTg---

PsCRN91 GaCccaTCgactGAcgAGTtTcGtTttcAgcggATTGAGgacGCGAagttCtTccTaccg

PsCRN172 -AtGGtgAtGgAGAAtGacGtt-ccTGcacctGAatcAGtgAAGCAGAttgCgggcAtG-

PsCRN91 tAcGGcaAgGcAGAAaGctGccattTGgtgtcGAgaaAGcaAAGCAGAgacCacaaAcGg

PsCRN172 --ATTtaCtccaTcCtgTCGgaAgaCAActag

PsCRN91 gaATTcgCcaagTaCgaTCGcgActCAAac--
